# Dental Pulp Stem Cell-Derived Extracellular Vesicles Mitigate Haematopoietic Damage after Radiation

**DOI:** 10.1007/s12015-020-10020-x

**Published:** 2020-08-04

**Authors:** Fanxuan Kong, Chu-Tse Wu, Panpan Geng, Chao Liu, Fengjun Xiao, Li-Sheng Wang, Hua Wang

**Affiliations:** 1 Department of Experimental Hematology, Beijing Institute of Radiation Medicine, No.27 Tai-ping Road, Hai-Dian District, Beijing, 100850 People’s Republic of China; 2 PLA Strategic Support Force Characteristic Medical Center, No.9 An-Xiang-Bei-Li, Chao-Yang District, Beijing, 100101 People’s Republic of China; 3 Beijing Key Laboratory for Radiobiology, No.27 Tai-ping Road, Hai-Dian District, Beijing, 100850 People’s Republic of China; 4 Binzhou Medical University, No.522 Huang-He-Third-Road, Yantai, 264003 People’s Republic of China

**Keywords:** Extracellular vesicles, Radiation injuries, Haematopoietic stem cells, Dental pulp stem cells, microRNA

## Abstract

Radiation therapy can cause haematopoietic damage, and mesenchymal stem cells (MSCs) derived extracellular vesicles (EVs) have been shown to reverse this damage. Our previous research showed that dental pulp stem cells (DPSCs) have a strong proliferation capacity and can produce abundant amounts of EVs to meet the requirements for use in vitro and in vivo. DPSCs derived EVs (DPSCs-EVs) are evaluated for their effect on reducing haematopoietic damage. Haematopoietic stem cell (HSC) numbers and function were assessed by flow cytometry, peripheral blood cell counts, histology and bone marrow transplantation. Epidermal growth factor (EGF) was used as a reference for evaluating the efficiency of EVs. miRNA microarray was employed to find out the changes of miRNA expression after cells being irradiated in vivo and the role they may play in mitigation the radiation caused injury. We observed the effect of DPSCs-EVs on promoting proliferation and inhibiting apoptosis of human umbilical vein endothelial cells (HUVECs) and FDC-P1 cells in vitro. We found that DPSCs-EVs and EGF could comparably inhibit the decrease in WBC, CFU count and KSL cells in vivo. We also verified that EVs could accelerate the recovery of long-term HSCs. In summary, DPSCs-EVs showed an apoptosis resistant effect on HUVECs and FDC-P1 cells after radiation injury in vitro. EVs from DPSCs were comparable to EGF in their ability to regulate haematopoietic regeneration after radiation injury in vivo. Radiation could alter the expression of some miRNAs in bone marrow cells, and EVs could correct these changes to some extent.

**Electronic supplementary material:**

The online version of this article (10.1007/s12015-020-10020-x) contains supplementary material, which is available to authorized users.

**Figure:**
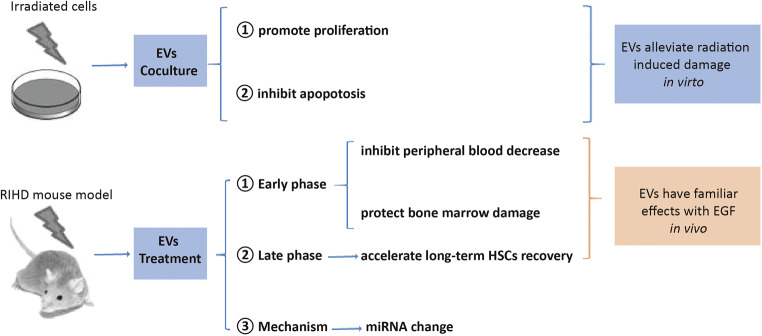
Graphical abstract

## Introduction

Since the first report that dogs exposed to a high dose of X-rays developed fatal haematopoietic toxicity [1], scientists have focused more attention on the devastating effects of ionizing radiation (IR) on human health. Since different tissues have different sensitivities to IR, radiation exposure could result in different levels of tissue injury. Compared to the gastrointestinal tract and nervous system, haematopoietic stem cells (HSCs) are more sensitive to IR. Over 100 cGy of total body irradiation (TBI) can affect the severity and duration of bone marrow suppression [2]. HSCs can be found in proximity to bone marrow sinusoidal vessels [3] and have the ability to self-renew and provide long-term multi-lineage haematopoiesis [4].

The HSC niche, which includes osteoblasts, sinusoidal endothelial cells (SECs), perivascular cells, mesenchymal stem cells (MSCs), adipocytes, CXCL12-abundant reticular (CAR) cells, macrophages, regulatory T cells (T_Reg_) and Schwann cells, plays a critical role in the microenvironment support for HSCs [2]. HSC maintenance and lineage differentiation are supported by stromal niches, and bone marrow stromal cells (BMSCs) are severely and permanently damaged by the pre-conditioning irradiation required for efficient HSC transplantation [5]. Radiation exposure also damages the HSC niche. It has been reported that transplantation of HSCs together with MSCs could enhance engraftment and improve bone marrow recovery from radiation injury [5, 6]. Lange et al. found that even transplantation of mouse bone marrow-derived mesenchymal stem cells (mMSCs) without additional stem cell transplantation improved the long-term survival of lethally irradiated recipients [7].

Therefore, not only should we pay attention to the HSCs themselves in haematopoietic regeneration but also to the HSC niche. Phuong Doan et al. found that systemic infusion of endothelial cells (ECs) could accelerate bone marrow HSC reconstitution and haematologic recovery in mice after radiation-induced myelosuppression, and epidermal growth factor (EGF) worked as a candidate endothelial cell-derived mediator of radioprotection of the haematopoietic system [8, 9].

It is assumed that most cells actively release diverse types of vesicles of endosomal and plasma membrane origin, called exosomes and microvesicles, respectively, into the extracellular environment. Here, we use the term EVs to describe all classes of extracellular membrane vesicles with a size of 30–1000 nm because most recent methods for purifying EVs result in a mixed population [10, 11]. EVs have been shown to participate in cell-to-cell communication. Many cell-derived materials, such as mRNAs, miRNAs, non-coding RNAs, proteins, lipids and DNA, have been found in small spherical membrane particles [12, 13]. In addition to biologically active components such as cytokines, extracellular vesicles (EVs) released by MSCs are promising candidates for mediating tissue regeneration [14]. Sicheng Wen et al. found that mesenchymal stromal cell-derived EVs could rescue radiation damage to marrow haematopoietic cells [15].

Dental pulp stem cells (DPSCs), which can be obtained from wisdom teeth, deciduous teeth, supernumerary teeth and impacted teeth, have shown rapid proliferation rates and are able to differentiate along typical mesodermal cell lineages such as chondrogenic, adipogenic and osteogenic lineages [16–22]. Compared with umbilical cord derived MSCs, DPSC have significant advantages for osteogenic differentiation, lower cell apoptosis, and senescence [23]. Our previous work showed that DPSCs could be easily genetically engineered [24], which implies that DPSCs could be a suitable source of EVs to meet their abundant need for in vivo and in vitro studies.

In this study, we sought to identify whether DPSCs-derived EVs (DPSCs-EVs) could mitigate haematopoietic damage after radiation.

## Methods

### Cells

Mouse bone marrow cell line FDC-P1 cells and human umbilical vein endothelial cells (HUVECs) were purchased from the National Infrastructure of Cell Line Resource (China). Primary DPSCs were generated and cultured using previously published methods [24]. Briefly, Normal human impacted third molars were collected from adults (19–29 years old) at the Dental Clinic of Beijing Stomatological Hospital. Tooth surfaces were cleaned and cut around the cementoenamel junction with sterilized dental fissure burst to reveal the pulp chamber. The pulp tissue was gently separated from the crown and root, cut into 1mm^3^ pieces, and then digested in collagenase and dispase (Sigma-Aldrich, St. Louis, MO) for 40 min at 37 °C. Tissue pieces were then cultured in a cell culture flask (Corning, NY) with alpha-modified Eagle’s medium (a-MEM; Gibco®; Thermo Fisher Scientific, Grand Island, NY) supplemented with 15% fetal bovine serum (FBS; Thermo Fisher Scientific) at 37 °C in 5% carbon dioxide. Cells were harvested on day 14. All cells in this study were used after four to six passages, and from the P4 cells, the conditioned media was changed to serum-free media (SANYL, Bejing Sanyouli Technology Advanced Co., China).

### Isolation and Characterization of Extracellular Vesicles

The isolation of EVs in this study was performed by differential ultracentrifugation. Briefly, the media from 4-day cultures of DPSCs were collected. Then, the media were centrifuged at 4 °C at 2000 rpm for 30 min to discard the dead cells and debris. EVs were separated by centrifugation at 4 °C at 110,000 g for 70 min (XPN-80, Beckman Coulter, USA). Then, phosphate buffered saline (PBS) was used to wash and resuspend the EV pellet, followed by a second centrifugation at 4 °C at 110,000 g for 70 min. The EV pellets were collected and resuspended in PBS. Generally, 25 mL conditioned media resulted in 200 μL of EVs. The purified EVs were stored at 4 °C for 2 weeks and at −80 °C for longer periods of time.

EVs were quantified with nanoparticle tracking analysis (NTA) (ParticleMetrix, Germany). The protein concentration in EVs was measured by Pierce™ BCA Protein Assay Kit (Thermo Scientific ™, USA) following the manufacture’s instruction. The EVs samples were then diluted to a suitable concentration according to their primary protein concentration and measured by the NTA machine for the first time. Then the diluted samples were diluted again to the final concentration of 5–10 × 10 ^7^ Particles/mL and measured again by the NTA machine to analysis their particle size distribution.

EVs were visualized by transmission electron microscopy (TEM) (H-7650, HITACHI, Japan). Briefly, EVs were diluted with PBS and dropped on the Formvar and copper-coated palladium grids. After the diluted EVs were dry, phosphotungstic acid was added to stain the EVs for image capture.

### Flow Cytometry Analysis

EV markers were detected by flow cytometry (FACSCalibur, BD, USA) following a previously published method [25]. Briefly, 5 μg purified EVs was incubated with 2 μL latex beads (Invitrogen, USA) for 15 min at room temperature. PBS was added to a final volume of 1 mL and the mix was incubated on a test tube rotator wheel overnight at 4 °C. A total of 110 μL of 1 M glycine was added, and incubated for 30 min at room temperature. The bead mix was microcentrifuged and the supernatant was removed. The bead pellet was resuspended in 1 mL PBS/0.5% BSA and washed three times. The beads were resuspended in 200 μL PBS/0.5% BSA. The coated beads were incubated with anti-exosomal protein antibody diluted in PBS/0.5% BSA for 30 min at 4 °C and washed. The antibody-stained exosome-coated beads were analysed on a flow cytometer. We detected the exosomal markers CD9, CD63, and CD81 (BD, USA) and the DPSC markers CD73, CD90, CD105, CD11b, CD19, CD34, CD45, and HLA-DR (eBioscience, USA).

### Animals

C57BL/6 (CD45.2+) mice were purchased from Beijing Vital River Laboratory Animal Technology Co., Ltd. and B6.SJL (CD45.1+) mice were donated by Beijing University. Mice were 6–8 weeks old at the time of all experiments, and biological variables such as age, sex, and weight were matched in all experiments.

### Haematopoietic Progenitor Cell Assays and Transplantation Assays

After ionizing radiation, C57BL/6 or B6.SJL mice were sacrificed, and BM cells were collected into RPMI 1640 Medium (Gibco, US) with 1% penicillin/streptomycin. Next, c-kit^+^Sca-1^+^lin^−^ (KSL) progenitor cells were measured following erythrocytes lysis as previously described [26] and were incubated with anti-lineage, anti-Sca-1, and anti-c-kit antibody cocktail (BD) containing antibodies against the following markers: Ly-6G, CD45R, CD3e, CD8a, TER-119, CD11b, NK-1.1 and CD19 (eBioscience, Biotin, USA).

Colony forming unit (CFU) assays for myeloid progenitor cells were performed following the manufacturer’s guidelines. Briefly, 2000 cells in 500 μL of MethoCult (StemCell, Canada) were cultured in 24-well plates (Corning, USA), and colonies were scored on day 7 by 2 investigators independently.

For the sublethal irradiation studies, 51 C57BL/6 mice were randomly divided into 4 groups: normal group (*n* = 12), control group (*n* = 13), EGF group (n = 13) and EVs group (n = 13). Except for those in the normal group, the mice were irradiated with 325 cGy total body irradiation (TBI) and then tail vein injection with PBS, EGF (PeproTech, USA), or EVs was performed starting at 6 h after irradiation. Injections were given at doses of 200 μL of diluted EGF/PBS (containing 10 μg EGF) or diluted EVs (containing approximately 2.5 × 10^9^ particles per dose) once daily from day 1 to 7. Mice were sacrificed on days 8, 15 or 30.

Competitive transplantation assays were performed using BM cells from 37 donor C57BL/6 mice (CD45.2^+^). The mice were randomly divided into 4 groups: normal group (*n* = 7), control group (*n* = 10), EGF group (n = 10), and EVs group (n = 10). Except for those in the normal group, the mice were irradiated with 700 cGy TBI, and then tail vein injection with PBS, EGF (PeproTech, USA), or EVs was performed starting at 6 h after irradiation. Injections were given at doses of 200 μL of diluted EGF/PBS (containing 10 μg EGF) or diluted EVs (containing approximately 2.5 × 10^9^ particles per dose) once daily from day 1 to 7. Mice were sacrificed at day 25 or when study endpoints were met according to protocols from Institutional Animal Care and Use Committee of the Beijing Institute of Radiation Medicine. The cells were collected from the femurs of mice in four groups. A total of 5 × 10^5^ donor cells with a competing dose of 1 × 10^5^ host mononuclear cells were transplanted into 20 B6.SJL recipient mice (CD45.1^+^) that had been lethally irradiated (800 cGy) 6 h before transplantation. The haemogram and weight of the recipient mice were measured every week until the study endpoint. CD45.2^+^ donor cell engraftment was measured at weeks 4, 6 and 8 post-transplantation by the calculating the percentage of CD45.2^+^ cells in peripheral blood.

### Apoptosis Assay

DPSCs were seeded in the lower chamber of Transwell plates (3470, Corning, USA) with a 0.4 μm filter that allowed the transport of EVs but not of cells. FDC-P1 cells were seeded in the upper chamber at 10000 cells/well after 200 cGy, 600 cGy or 1000 cGy irradiation and co-cultured with DPSCs for 3 days. Apoptotic cells were identified by an Annexin V-APC Apoptosis Analysis Kit (Tianjin Sungene Biotech) according to the manufacturer’s instructions.

### Cell Proliferation Assay

Cell proliferation was measured by Cell Proliferation Dye eFluor® 670 (Dye 670) (Invitrogen, CA, USA) according to the manufacturer’s instructions. In principle, the Dye 670 could bind to any cellular protein containing primary amines, and as cells divide, the dye is distributed equally between daughter cells that can be measured as successive halving of the fluorescence intensity of the dye. We observed the cells for 96 h and used the flow cytometry to analysis the proliferation index. In the co-culture experiment, HUVECs were co-cultured with DPSC-EVs for 3 days, and the cell proliferation index was also measured by Dye 670.

FDC-P1 cells were co-cultured with EVs and seeded in 96-well plates at 1000 cells/well. Three groups were divided: High Dose-EVs group (H-EVs), one FDC-P1 cell co-cultured with about 9 × 10^4^ EV particles, Low Dose-EVs group(L-EVs), one FDC-P1 cell co-cultured with about 1.8 × 10^4^ EV particles, and control group, FDC-P1 cells co-cultured with an equal volume of PBS. Cell proliferation was measured by a Cell Counting Kit-8 (CCK8) (Bimake, China) according to the manufacturer’s instructions. CCK8 allows convenient assays using WST-8 (2-(2-methoxy-4-nitrophenyl)-3-(4-nitrophenyl)-5-(2,4-disulfophenyl)-2H-tetrazolium, monosodium salt), which produces a water-soluble formazan dye upon bioreduction in the presence of an electron carrier, 1-Methoxy PMS. The proliferation index was measured at 0 h, 24 h, 48 h and 72 h after the co-culture.

### miRNA Analysis

In order to detect the micro-RNA change in mouse bone marrow after the radiation and the EVs injection, we select three samples for each group (normal group, control group and EVs group) 25 days after the C57BL/6 mice being irradiated in the competitive transplantation assays. Then, we send these samples to CNKINGBIO Corporation (Beijing, China) for miRNA microarray detection.

### Statistical Analysis

Graphs are presented as the mean ± SEM (standard error of the mean). GraphPad Prism version 6 (GraphPad Software, USA) was used for statistical analysis. Unpaired Student’s t test was used to evaluate the statistical significance, and statistical significance was indicated as follows: *P ≤ 0.05, **P ≤ 0.01 and ***P ≤ 0.001. A *P* value ≤0.05 was considered statistically significant.

## Results

### EV Isolation and Characterization

**Fig. 1:**
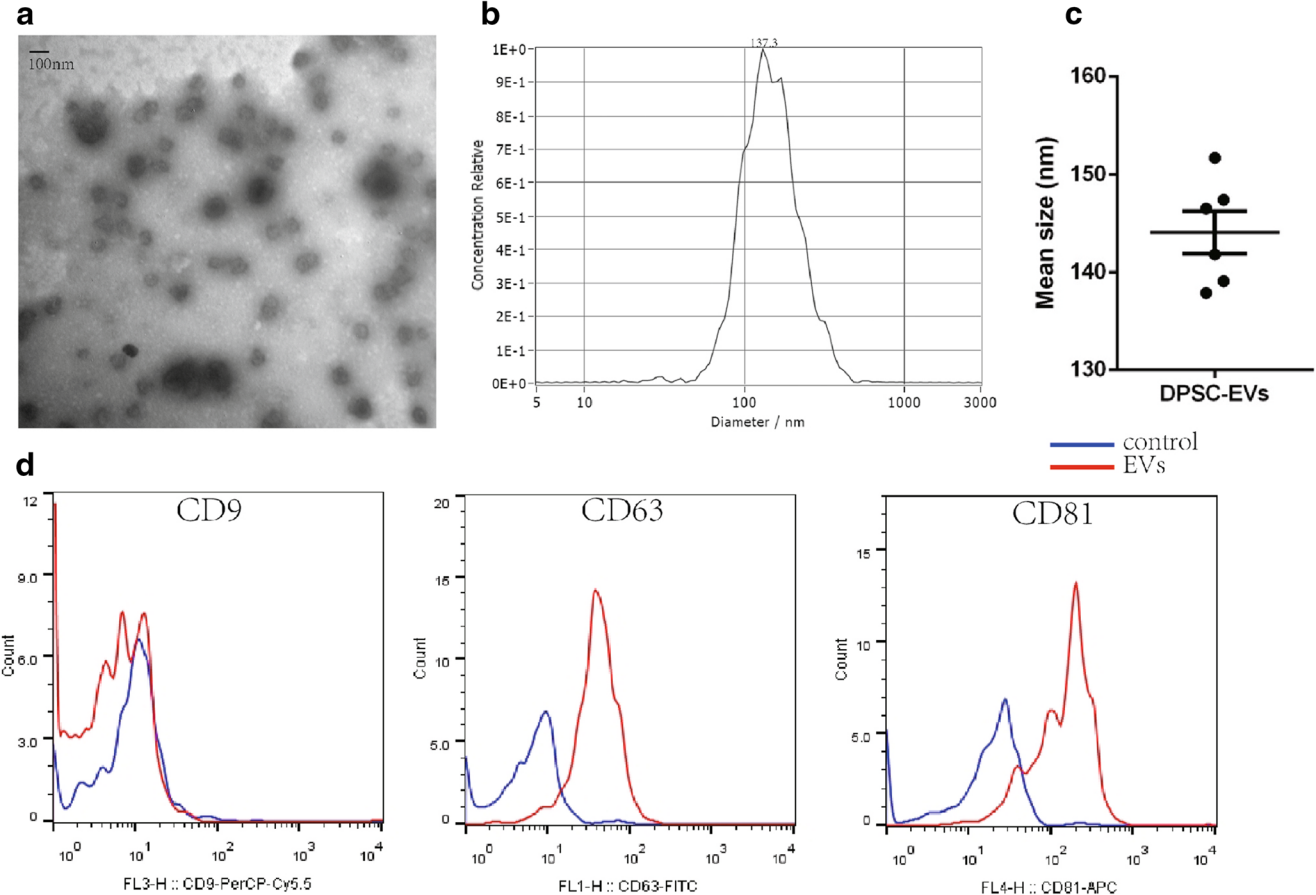
Characterization of dental pulp stem cell-derived extracellular vesicles (EVs). (**a**). Transmission electron microscopy image of DPSCs-EVs. Scale bar, 100 nm. (**b**). Left, size distribution profiles for DPSCs-EVs as measured by Nanoparticle Tracking Analysis (ParticleMetrix, Germany). Right, mean size of EVs. The mean size was 144 nm. *N* = 6. (**c**). Flow cytometry analysis of CD9, CD63, and CD81 on EVs. Data are shown as the means±SEMc

The EVs were isolated by differential ultracentrifugation. Some EVs were seen as round- or cup-shaped bilayer structures with varied sizes by transmission electron microscopy (TEM) (Fig. 1a). We used the Nanoparticle Tracking Analysis (ParticleMetrix, Germany) to measure the size distribution profiles for DPSC-EVs and found a single peak for the EVs isolated by differential ultracentrifugation (Fig. 1b). The mean size was 144.1 ± 2.2 nm (Fig. 1c). Flow cytometry analysis for the exosomal markers CD9, CD63, and CD81 was performed, and the EVs stained positive for CD63 and CD81 (Fig. 1d). Considering that the EVs were isolated from a DPSC culture, we also detected DPSC markers. Flow cytometry analysis showed that CD105, CD90, and CD73 staining was positive and that HLA-DR, CD45, CD34, CD11b and CD19 staining was negative (Supplementary Fig. 1), which is consistent with the markers of DPSCs [24].

### EVs Promoted HUVECs Proliferation

**Fig. 2:**
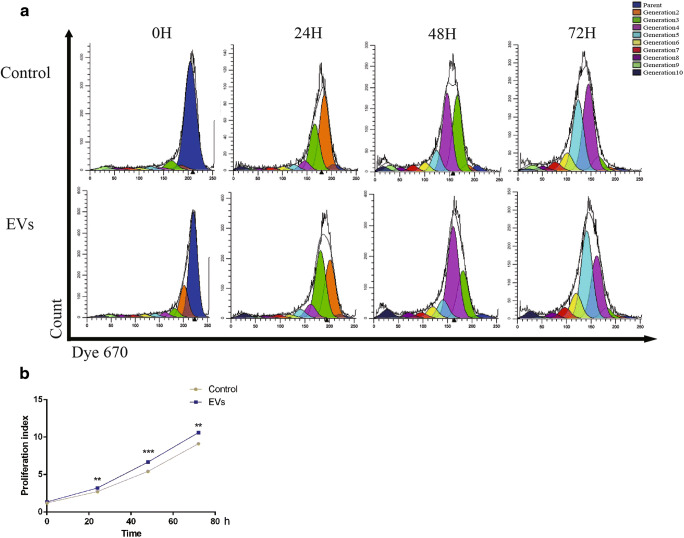
DPSCs-EVs promoted the proliferation of HUVECs. (**a**). Flow cytometry analysis for proliferation after HUVECs were co-cultured with PBS or EVs. Top: the HUVECs were co-cultured with PBS and the fluorescence intensity of the Dye 670 at 0 h, 24 h, 48 h,72 h after the bind of dye and cell protein. Bottom: the HUVECs were co-cultured with EVs and the fluorescence intensity of the Dye 670 at 0 h, 24 h, 48 h,72 h after the bind of dye and cell protein. The blue bar represented Parent which meant the cells had not divided, the orange bar represented Generation 2 which meant the cells had divided once, the green bar represented Generation 3 which meant the cells had divided twice, and so on. (**b**). Statistical analysis of the control and EVs groups. *N* = 3. **P < 0.01, ***P < 0.001

HUVECs were co-cultured with EVs for 3 days, and the HUVECs proliferation was measured by Dye 670 and detected by flow cytometry (Fig. 2a). Statistical analysis showed that the EVs group had a higher proliferation index than the control group at 24 h, 48 h and 72 h (Fig. 2b), which indicated that co-culture with EVs could promote HUVECs proliferation.

### Effects of EVs on FDC-P1 Cells Proliferation and Apoptosis Caused by Radiation

**Fig. 3:**
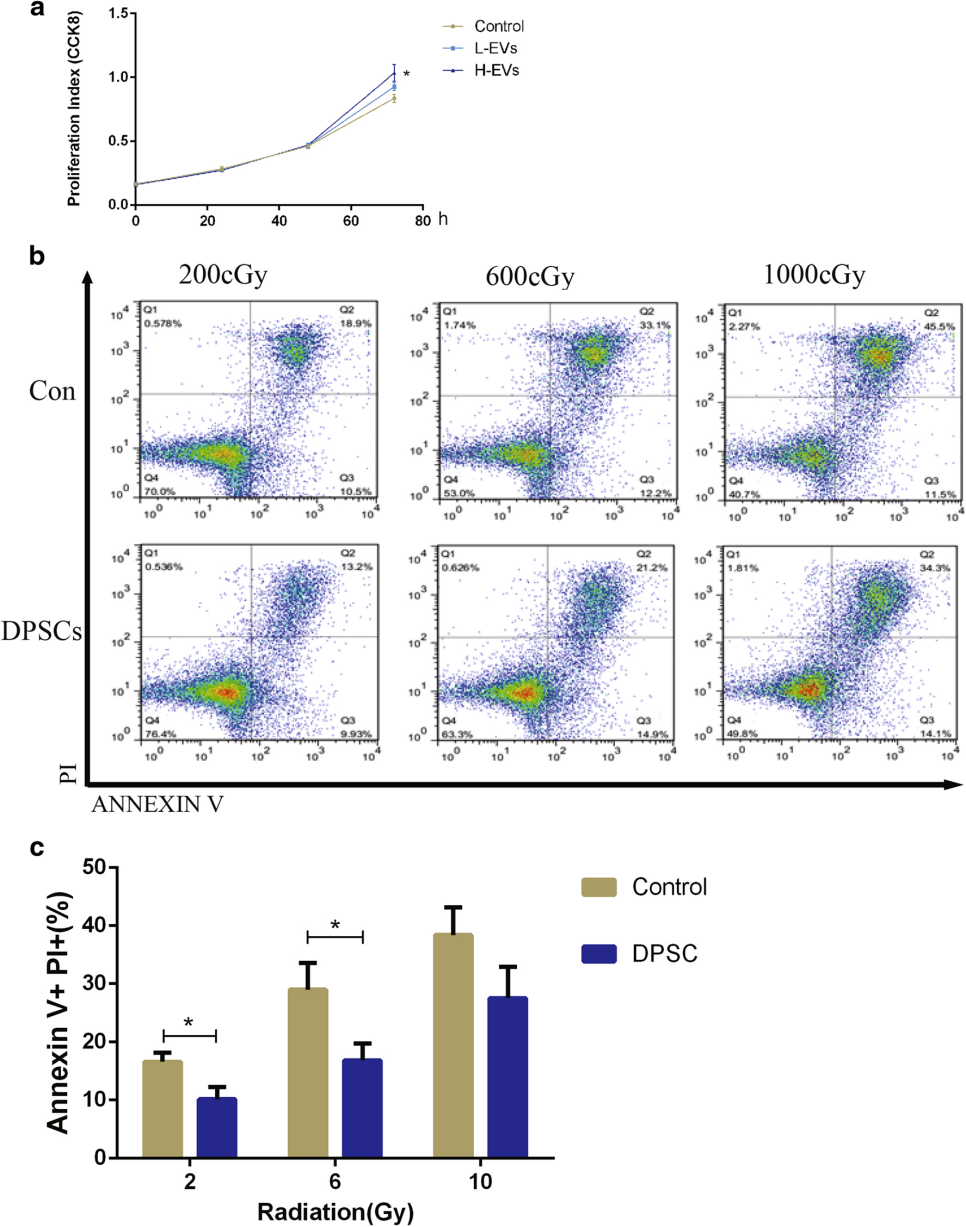
Co-culture with DPSCs-EVs or DPSCs could promote the proliferation or inhibit the apoptosis of FDC-P1 cells in vitro. (**a**). FDC-P1 cells co-cultured with EVs at different dose. *P < 0.05 (con vs H-EVs). *N* = 4. L-EVs, low dose of EVs; H-EVs, high dose of EVs. (**b**). FDC-P1 cells were irradiated with 200 cGy, 600 cGy or 1000 cGy before co-cultured with DPSCs, and apoptosis was measured by flow cytometry. (**c**). Statistical analysis of the difference between control and DPSCs groups. *P < 0.05. N = 3. Data are shown as the means ± SEM. Student’s t test (2-tailed with unequal variance) was applied to these data

We co-cultured FDC-P1 cells with different concentration of DPSCs-EVs and measured cell proliferation by CCK8. The results demonstrated that after co-culture for 72 h, the difference between the control group and the H-EVs group was statistically significant, but there were no statistical significance between L-EVs group and control group(Fig. 3a). These results indicate that the effects of EVs on FDC-P1 cells proliferation may be dependent on the concentration of EVs.

FDC-P1 cells were irradiated with 200, 600 or 1000 cGy and then divided into two groups: the control group, which was cultured alone; the EVs group, which was co-cultured with DPSCs. The apoptotic cells were measured by flow cytometry (Fig. 3b). The results showed that the percentage of apoptotic cells in the control group was 16.6 ± 1.6%, 29.0 ± 4.6%, and 38.4 ± 4.8% after 200, 600 or 1000 cGy radiation, respectively. However, in the DPSCs group, the numbers were 10.1 ± 2.1%, 16.8 ± 2.9% and 27.5 ± 5.4%, respectively (Fig. 3c). The results indicated that co-culture with DPSCs could inhibit the apoptosis of FDC-P1 cells induced by radiation.

### Effects of EVs on Haematopoietic Regeneration In Vivo

**Fig. 4:**
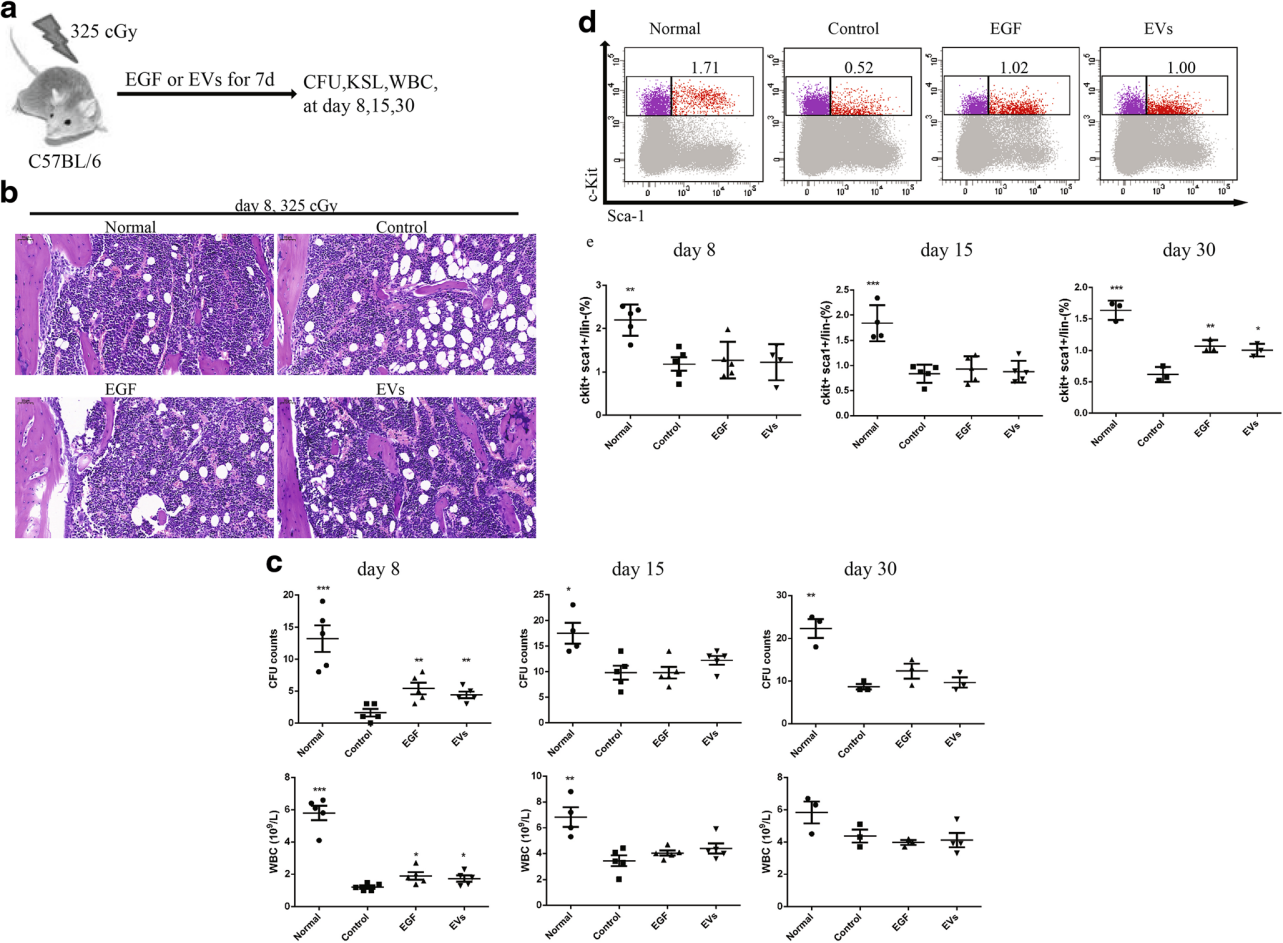
Extracellular vesicles (EVs) mitigated radiation injury to haematopoietic stem and progenitor cells after 325 cGy radiation. (**a**). Schematic diagram of the experimental design. (**b**). Light micrographs of haematoxylin and eosin-stained femurs at day 8 after 325 cGy radiation. Scale bar, 50 mm. (**c**). CFU and WBC statistics analysis of different groups. N = 3–5. (**d**). Representative flow cytometric analysis of bone marrow c-Kit^+^Sca-1^+^ cells within the Lin^−^ gate (KSL) of different groups at Day 30. The number above the right box shows the percentage of c-Kit^+^Sca-1^+^ cells within the Lin^−^ population. (**e**). Quantification of percentage KSL. N = 3–5. Data are shown as the means ± SEM. Student’s t test (2-tailed with unequal variance) was applied to these data. *P < 0.05, **P < 0.01, ***P < 0.001 vs the control group

To determine whether EVs could promote haematopoietic regeneration in vivo, we measured the WBC numbers, CFU and KSL in irradiated mice treated with EVs or PBS and compared these results to those in EGF-treated mice (Fig. 4a). Femurs were stained with haematoxylin and eosin, and we noted that there were numerous vacuoles and decreased marrow cellularity in the control group at day 8 (Fig. 4b). After 325 cGy TBI at day 8, the WBC number of peripheral blood and CFU counts of bone marrow cells in the control group decreased significantly compared to the normal group. In addition, the EGF and EVs groups inhibited this decrease compared to the control group, but there was no statistically significance between these two groups (Fig. 4c). We used flow cytometry to detect the bone marrow KSL cells (Fig. 4d). The percentage of c-Kit^+^Sca-1^+^ cells within the Lin^−^ population in the control group continuously declined from day 8 to day 30. Importantly, this percentage in the EGF, and EVs groups decreased at day 8 and day 15, but the decline was inhibited at day 30 compared to that in the control group. In addition, there were no differences between the EGF and EVs groups, indicating that EVs and EGF could comparably promote haematopoietic regeneration (Fig. 4e).

### EVs Accelerated the Recovery of Long-Term HSCs

**Fig. 5:**
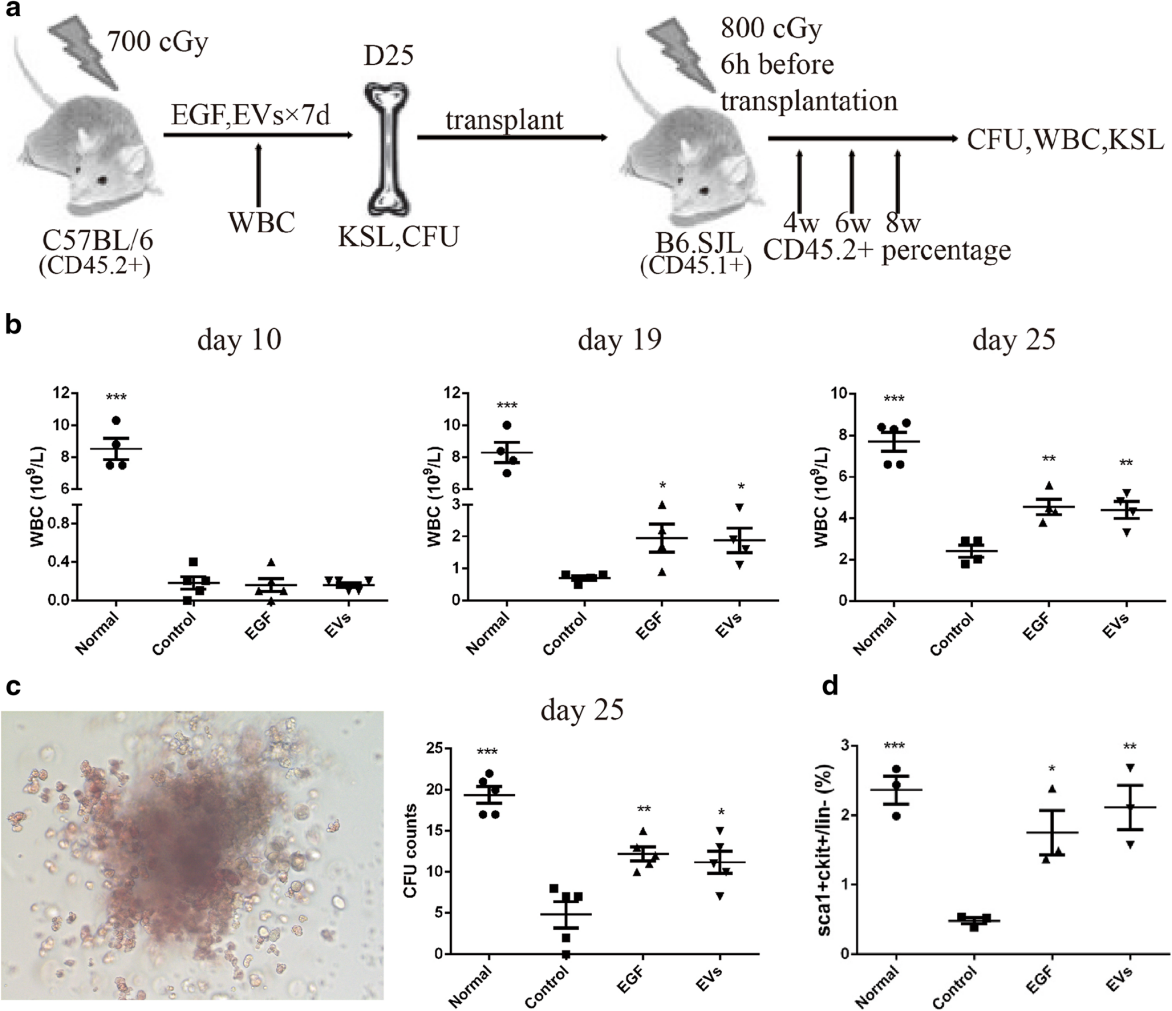
Extracellular vesicles (EVs) improved the recovery of LT-HSCs. (**a**). Schematic diagram of the experimental design. (**b**). WBC statistics analysis of different groups. (**c**). CFU analysis at Day 25. The left figure shows the CFU culture for 7 days after 24 days post-radiation. The right one is the CFU statistics analysis of different groups. (**d**). KSL statistical analysis after 24 days of radiation. N = 3–5. Data are shown as the means ± SEM. Student’s t test (2-tailed with unequal variance) was applied to these data. *P < 0.05, **P < 0.01, ***P < 0.001 vs the control group

To measure the effect of EVs on long-term HSCs (LT-HSCs), we conducted competitive transplantation assays (Fig. 5a). We irradiated C57BL/6 (CD45.2^+^) mice with 700 cGy and then treated them with EGF, EVs or PBS (control) by intraperitoneal injection daily from day 1 to day 7. We noticed that the WBC number declined significantly in the control, EGF and EVs groups at day 10. However, at day 19, the WBC showed a better recovery in the EGF and EVs groups compared with the control group, and at day 25, the difference was more significant (Fig. 5b).

At day 25, the C57BL/6 mice were sacrificed, and the CFUs were cultured for 7 days. The CFU counts indicated that EGF or EVs treatment could inhibit the CFU decline (Fig. 5c). Flow cytometric analysis showed that the percentage of c-Kit^+^Sca-1^+^ cells within the Lin^−^ population in the control group decreased to approximately 0.48 ± 0.05% at Day 25, and those in the EGF and EVs groups were approximately 1.75 ± 0.32% and 2.12 ± 0.32%, respectively (Fig. 5d). There were no statistically significant differences between the EGF and EVs groups.

**Fig. 6:**
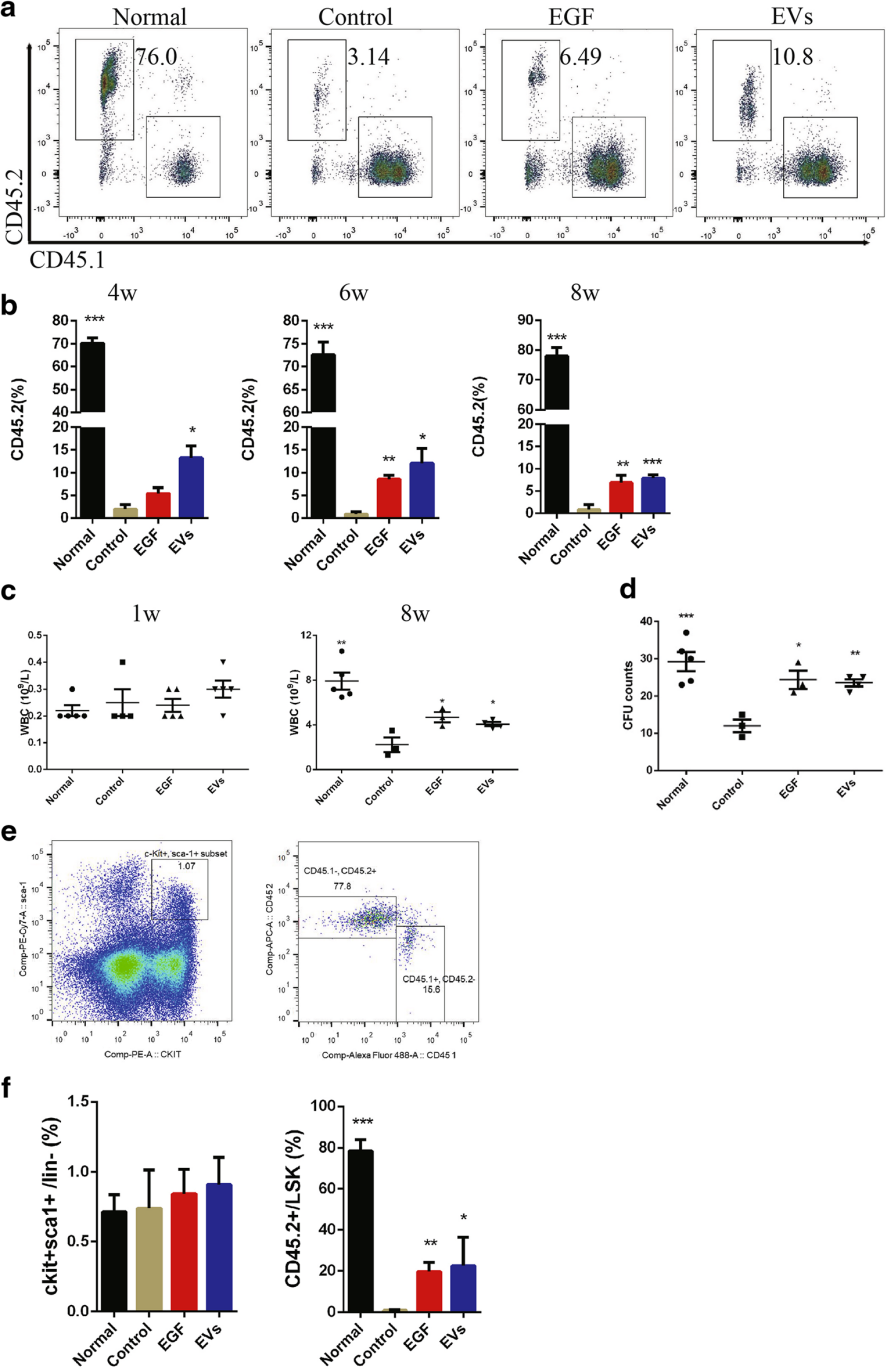
EVs treatment promote haematopoietic regeneration of B6.SJL recipient mice after 800 cGy radiation. (**a**). Representative flow cytometry analysis of peripheral CD45.2^+^ cells of different groups at Week 4, and the number shows the percentage of CD45.2^+^CD45.1^−^ cells. (**b**). Quantification of the percentage of CD45.2^+^ cells. (**c**). WBC statistical analysis of irradiated mice after cell transplantation. (**d**). CFU statistics analysis at 8 weeks after transplantation. (**e**). Representative flow cytometric analysis of KSL at Week 8. The left figure shows the percentage of c-Kit^+^Sca-1^+^ cells within the Lin^−^ population. The right figure shows the percentage of CD45.2^+^CD45.1^−^ cells or CD45.2^−^CD45.1^+^ cells. (**f**). The KSL and CD45.2^+^ cell percentages in the KSL statistical analysis at 8 weeks after transplantation. N = 3–5. Student’s t test (2-tailed with unequal variance) was applied to these data. *P < 0.05, **P < 0.01, ***P < 0.001 vs the control group

We measured the CD45.2^+^CD45.1^−^ cells in B6.SJL mice, which had been irradiated with 800 cGy before transplantation, in the peripheral blood at 4, 6 or 8 weeks after transplantation (Fig. 6a). The percentage of CD45.2^+^CD45.1^−^ cells was approximately 1.96 ± 0.99%, 0.88 ± 0.51% and 0.84 ± 0.63% at Week 4, 6 and 8, respectively, in the control group. However, the number in the normal group was over 70% at different times. For treatment with EGF or EVs, the percentage of CD45.2^+^CD45.1^−^ cells improved compared to that in the control group. There were still no statistically significant differences between the EGF and EVs groups (Fig. 6b). Therefore, transplanted EGF- or EVs-treated donor cells would be more likely to survive in the irradiated mice.

B6.SJL (CD45.1^+^) mice were irradiated 800 cGy before cell transplantation. The WBC in all groups decreased dramatically at week 1. However, the EGF and EVs groups showed better WBC recovery, with 4.7 ± 0.5 × 10^9^/L in the EGF group and 4.1 ± 0.2 × 10^9^/L in the EVs group at Week 8, compared with that in the control group, which was 2.2 ± 0.7 × 10^9^/L (Fig. 6c). EVs- and EGF-treated cells could comparably promote peripheral blood WBC recovery in recipient mice.

The B6.SJL (CD45.1^+^) mice were sacrificed 8 weeks after cell transplantation. We isolated the bone marrow cells and measured the CFUs and KSL cells. We found that the CFU counts in the control group decreased significantly, and EGF and EVs could promote haematopoietic regeneration (Fig. 6d). We also compared the percentage of c-Kit^+^Sca-1^+^ cells within the Lin^−^ population and the percentage of CD45.2^+^ cells within KSL cells. Interestingly, there were no significant differences in c-Kit^+^Sca-1^+^ cell percentages among the four groups. However, the CD45.2^+^ percentage in KSL cells was approximately 78.5 ± 5.3% in the control group, 1.0 ± 0.2% in the control group, 19.7 ± 2.6% in the EGF group and 22.5 ± 7.0% in the EVs group. These results indicated that EVs and EGF could improve the recovery of LT-HSCs.

### Some miRNA Expression Changes after the Bone Marrow Cells Being Radiated and EVs Injection

**Fig. 7:**
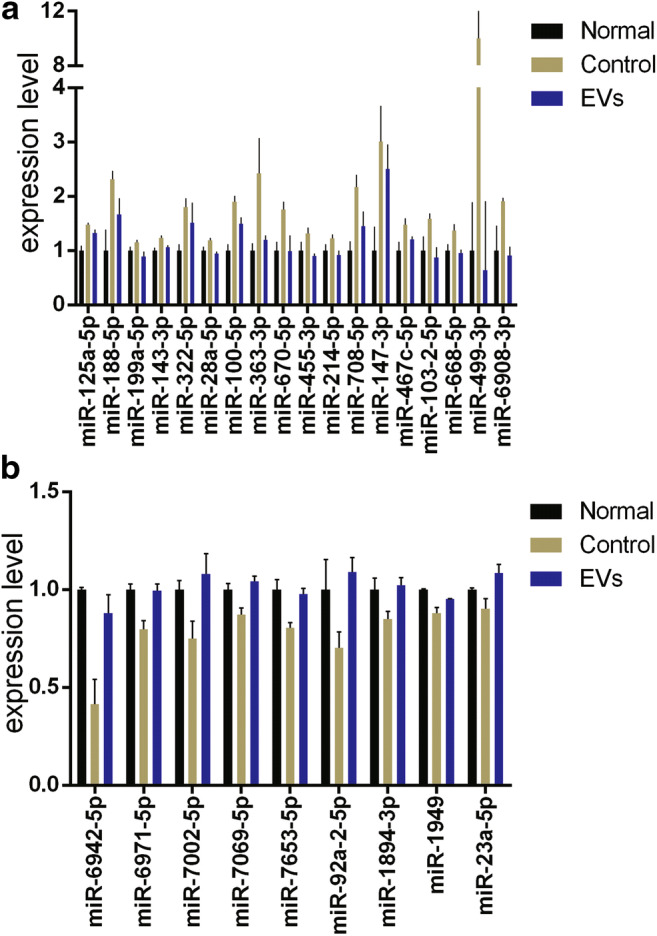
The expression of miRNA in bone marrow cells 25 days after the mice being irradiated with 700 cGy TBI treated with daily EVs (EVs group) injection or PBS (control group) for 7 days. (**a**). The miRNAs which increased after the radiation and EVs injection could alleviate this increase. (**b**). The miRNAs which decreased after the radiation and EVs injection could alleviate this decrease. Data are shown as the means±SEM

It has been found that EVs contain large amounts of miRNAs and can serve as vehicles to transfer miRNAs to recipient cells, where the exogenous miRNAs can alter the gene expression and bioactivity of recipient cell. In this study, the miRNA expression changes in bone marrow cells were analysed after the C57BL/6 mice being irradiated. We found that the expression of miR-125a-5p, miR-188-5p, miR-143-3p, miR-322-5p, miR-28a-5p, miR-100-5p, miR-363-3p, miR-670-5p, miR-455-3p, miR-214-5p, miR-708-5p, miR-147-3p, miR-467c-5p, miR-103-2-5p, miR-668-5p, miR-499-3p and miR-6908-3p were increased after the irradiation. Meanwhile, the EVs daily injection for 7 days after the irradiation could alleviate these increases (Fig. 7a). The expression of miR-6942-5p, miR-6971-5p, miR-7002-5p, miR-7069-5p, miR-7653-5p, miR-92a-2-5p, miR-1894-3p, miR-1949 and miR-23a-5p were decreased after the irradiation. However, the EVs injection could promote these expression (Fig. 7b).

## Discussion

We have developed methods to isolate and quantify EVs from DPSCs. Unlike BMMSCs, DPSCs are obtained from deciduous teeth, wisdom teeth, supernumerary teeth and impacted teeth, which causes nearly no damage to the donors. In addition, DPSCs have a stronger proliferation ability than BMMSCs, and it is possible to harvest over 6 × 10^9^ DPSCs at P6 from one dental pulp. DPSCs are also suitable for genetic engineering which may provide a broader application prospects.

We found that DPSCs-EVs could promote the proliferation of HUVECs in vitro. Then, we co-cultured FDC-P1 cells, a mouse bone marrow cell line, with DPSCs-EVs and found that EVs could also promote the proliferation of FDC-P1 cells. Importantly, when radiated FDC-P1 cells co-cultured with DPSCs in vitro, radiation-induced apoptosis was inhibited.

Many researchers have demonstrated that the administration of MSCs can protect and reverse radiation damage to the bone marrow [7, 27]. Our previous work also revealed that MSCs derived from umbilical cord could attenuate radiation-induced lung injury [28]. An increasing number of researchers have realized that the therapeutic effect of MSCs on radiation injury may not mainly rely on the transplant cells homing to the damaged tissue because after intravenous injection of MSCs, there is only a small number of MSCs found in the damaged area, but there is a significant increase in haematopoietic recovery after irradiation [7, 27]. Wen et al. found that EVs derived from MSCs can rescue radiation damage to murine marrow haematopoietic cells [15]. Piryani et al. proved that EVs derived from endothelial cells could mitigate radiation-induced haematopoietic injury [29]. Therefore, MSCs engraftment or repopulation of target cells by MSCs might not be required.

EVs can transfer proteins, lipids and nucleic acids between cells, thereby influencing various physiological and pathological functions of both recipient and parent cells [30]. There are three subtypes of EVs, namely, exosomes, shedding microvesicles or ectosomes and apoptotic bodies [31]. Wen et al. proved that the use of exosomes and microvesicles together showed the largest effect on the reversal of radiation damage [15]. Therefore, we used ultracentrifugation to isolate EVs and did not distinguish the effect of exosomes or microvesicles in this study. We also investigated the changes in microRNA expression after the mice being radiated and EVs treatment. High-throughput sequencing showed that microRNA expression in mouse bone marrow cells changed after irradiation, and EV treatment could alleviate the increases of miR-125a-5p, miR-143-3p, miR-199a-5p and miR-455-3p and decreases of miR-23a-5p and miR-1894-3p. Some roles of these microRNA have been revealed, such as the miR-125a-5p can affect cell migration and modulate sensitivity to ionizing radiation [32], the miR-143-3p increases in exosome-like EVs from irradiated whole blood samples and contributes to early stages of radiation-induced genomic instability [33, 34], the miR-199a-5p works as a novel and unique regulator of radiation induced-autophagy [35], and the miR-455-3p regulates cell growth, metastasis, and glycolysis in hepatocellular carcinoma [36]. The miR-23a-5p blocks the effect of the acceleration of cell proliferation and alleviation of cell cycle arrest [37], and the miR-1894-3p works as an important antimetastatic miRNA in lung metastasis [38].

The underlying mechanisms may need further investigation. These previous discoveries are consistent with what we found and will help us better understand the mechanism of EVs in treating radiation injury.

In this study, we used EGF as a positive therapeutic agent because it has been proved that systemic administration of EGF accelerated the recovery of LT-HSCs and improved the survival of mice after radiation-induced myelosuppression [9]. We demonstrated that DPSCs-derived EVs could accelerate the WBC recovery and inhibit the decline of CFU and c-Kit^+^Sca-1^+^ cells after mice being radiated. The efficacy of EVs is comparable to that of EGF for haematopoietic regeneration, and in some measurements, especially LT-HSCs measured by competitive transplantation assay, the EVs performed even better than EGF. We took more attention to KSL progenitor cells and analyzed Ly-6G, CD45R, CD3e, CD8a, TER-119, CD11b, NK-1.1 and CD19 negative lin- cells in our experiment. However, the characterization of the haematopoietic cell sub-populations, including CD13/14, CD19, CD3, CD41 and CD71/glicophorin A, is very important, and EV treatment might favor regeneration of specific haematopoietic cell lineage and this will be a very worthwhile study. We will pay attention to the changes of these haematopoietic cell sub-population and megakaryocyte-erythroid progenitor, granulocyte-monocyte progenitor and common lymphoid progenitor after the EV treatment in our following research.

Due to the feasibility and convenience of genetic engineering of DPSCs, we could introduce miRNAs into the DPSCs to better understand the role of miRNAs played in radiation injury mitigation and may improve the therapeutic effect further in future.

## Conclusions

In conclusion, DPSCs-derived EVs could improve haematopoietic regeneration after radiation injury in vitro and in vivo, especially for the LT-HSCs. Besides, DPSCs-EVs had an effect on cell proliferation and apoptosis inhibition in vitro. The miRNA in EVs may play an important role in mitigation the radiation injury. This is the first report that EVs derived from DPSCs could be used in radiation injury. These findings might introduce a new therapy for radiated patients in the future.

## Electronic supplementary material

Supplementary Fig 1 Flow cytometry analysis of DPSCs markers on EVs (JPG 1045 kb)

## Abbreviations

**BMSC**: bone marrow stromal cell
**CAR**: CXCL12-abundant reticular
**CFU**: colony forming unit
**DPSC**: dental pulp stem cell
**EC**: endothelial cell
**EGF**: epidermal growth factor
**EV**: extracellular vesicle
**HSC**: haematopoietic stem cell
**HUVEC**: human umbilical vein endothelial cell
**IR**: ionizing radiation
**KSL**: c-kit^+^Sca-1^+^lin^−^
**LT-HSC**: long-term haematopoietic stem cell
**mMSC**: marrow-derived mesenchymal stromal cell
**MOI**: multiplicity of infection
**MSC**: mesenchymal stromal cell
**NTA**: nanoparticle tracking analysis
**PBS**: phosphate buffered saline
**SEC**: sinusoidal endothelial cells
**SEM**: standard error of the mean
**TBI**: total body irradiation
**TEM**: transmission electron microscopy
**Treg**: regulatory T cells
**WBC**: white blood cell
