# Supplementary figures and images for: Dental Pulp Stem Cell-Derived Extracellular Vesicles Mitigate Haematopoietic Damage after Radiation
Source: Stem Cell Rev Rep. 2020 Aug 4;17(2):318–31. doi: 10.1007/s12015-020-10020-x (PMC8036185; doi:10.1007/s12015-020-10020-x)

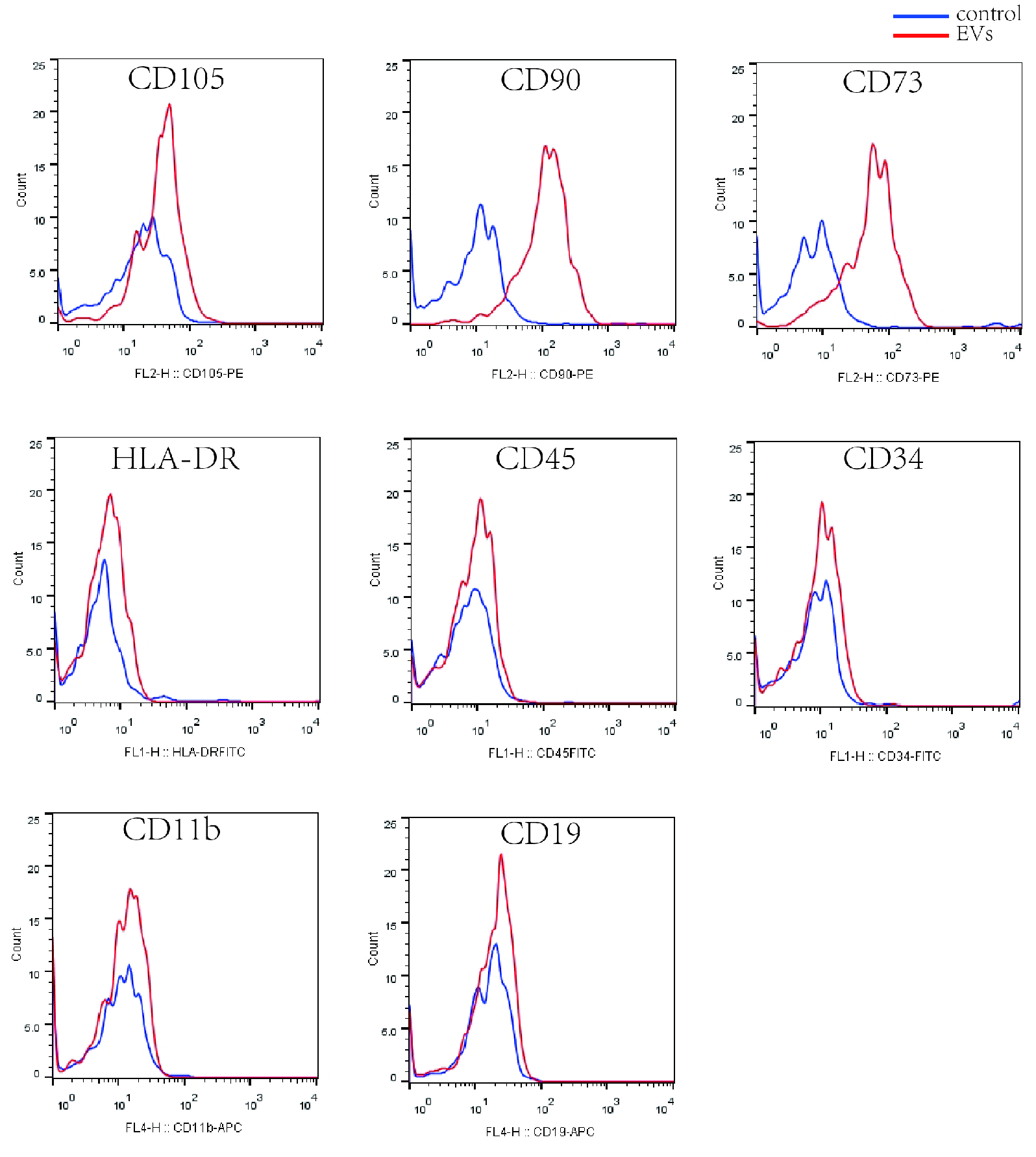

Supplement: Supplementary file 1 — Flow cytometry analysis of DPSCs markers on EVs (JPG 1045 kb) [file 12015_2020_10020_MOESM1_ESM.jpg]
